# Non-tuberculous mycobacterial infections in mainland China and Taiwan: a systematic review and meta-analysis of epidemiology, species distribution, and drug resistance (2013–2024)

**DOI:** 10.3389/fpubh.2025.1676715

**Published:** 2025-12-05

**Authors:** Xinru Xu, Yanyi Lei, Li Zheng

**Affiliations:** Department of lmmunology and Key Laboratory of Tropical Translational Medicine of Ministry of Education, College of Basic Medical Sciences, Hainan Medical University, Haikou, Hainan, China

**Keywords:** NTM (nontuberculous mycobacteria), China, meta-analysis, prevalence, drug resistance

## Abstract

**Introduction:**

Non-tuberculous mycobacteria (NTM) represent an emerging public health threat in China. Despite evidence of rising NTM prevalence and significant regional variation, comprehensive and up-to-date nationwide data on epidemiology, species distribution, and drug resistance patterns have been lacking. This study aimed to systematically evaluate the prevalence, geographical and temporal trends, species composition, and antimicrobial resistance of NTM in mainland China and Taiwan from 2013 to 2024.

**Methods:**

Adhering to PRISMA guidelines, this systematic review and meta-analysis (PROSPERO: CRD42024540102) included 43 studies (2013–2024) encompassing 17,959 NTM isolates from 225,733 suspected TB patients. Stata software was used for meta-analysis with a random-effects model; publication bias was assessed via Egger’s test and funnel plots.

**Results:**

The pooled NTM prevalence among suspected tuberculosis patients was 11.27% (95% CI: 9.89–12.65%), with marked geographic variation: highest in northeast (24.18%) and southeast coastal regions (12.83%), and lowest in southwest (2.30%). Slowly growing mycobacteria accounted for 68.07% of isolates, dominated by *Mycobacterium avium* complex (especially *M. intracellulare*); rapidly growing mycobacteria (26.57%) were more prevalent in southern provinces, with *M. abscessus* predominant. An overall upward trend in NTM isolation was observed from 2009–2021, followed by a decline in 2022–2023. Widespread resistance to first-line antituberculosis drugs was universal, whereas clarithromycin, amikacin, linezolid, and clofazimine retained good activity.

**Conclusion:**

NTM prevalence in China has continued to rise over the past decade and now exceeds many global estimates, with pronounced coastal–southern predominance. The persistently high resistance to standard antituberculosis drugs underscores the urgent need for standardized diagnostic protocols, mandatory NTM reporting, enhanced surveillance networks, and region-tailored treatment guidelines.

**Systematic review registration:**

https://www.crd.york.ac.uk/prospero/, identifier CRD42024540102.

## 1 Introduction

Non-tuberculous mycobacteria (NTM) refer to mycobacteria other than the *Mycobacterium tuberculosis* complex and *Mycobacterium leprae*. These organisms are ubiquitous in natural environments, including water, soil, and dust. To date, over 200 species of NTM have been identified (1). While many NTM species are benign, certain ones act as opportunistic pathogens, causing infections in humans that primarily affect the lungs, lymph nodes, bones, joints, skin, and soft tissues; they can also progress to systemic disseminated diseases. Pulmonary NTM disease remains the most common clinical manifestation (2). Initially, it was believed that humans contract NTM infections primarily through environmental exposure, such as contact with soil, water, and aerosols, with no transmission occurring between animals and humans or between humans. However, recent studies utilizing whole-genome sequencing have suggested that certain NTM species, such as *M. abscessus*, may be transmissible between humans (3), challenging earlier assumptions and highlighting the need for further investigation. China, a developing nation with a significant tuberculosis (TB) burden, reported an estimated 748,000 new TB cases in 2022, with an incidence rate of 52 per 100,000. According to the World Health Organization’s 2023 Global Tuberculosis Report, China ranks third globally in TB burden, contributing 7.1% to the global TB incidence, following Indonesia (10%) and India (27%) (4). The same report estimated 30,000 TB-related deaths in China, corresponding to a mortality rate of 2.0 per 100,000 (4). In addition to this substantial TB burden, China is witnessing a rising prevalence of NTM pulmonary disease, particularly in its southern and coastal regions. A nationwide study conducted in 2020 revealed that slow-growing NTM species are distributed throughout the country, while rapidly growing species predominate in southern and coastal areas (5). Large-scale epidemiological data on non-tuberculous mycobacterial (NTM) disease remain scarce in China. However, data from previous national tuberculosis (TB) epidemiological surveys in the country show that the isolation rate of NTM has increased from 4.3% in 1979 to 11.1% in 2000, and further to 22.9% in 2010—reflecting a clear upward trend in NTM disease prevalence across China (3). Existing literature highlights significant regional variability in NTM disease distribution across China, with higher prevalence observed in coastal and southern regions compared to inland and northern areas, and in temperate climates compared to colder zones (6). This geographical pattern mirrors the global trend of increasing NTM isolation and incidence rates. Most NTM strains exhibit intrinsic resistance to first- and second-line anti-TB drugs, resulting in suboptimal treatment outcomes, higher recurrence rates, and elevated mortality. Additionally, the clinical presentation of NTM infections is often atypical and closely resembles that of TB, frequently leading to misdiagnosis. This overlap complicates TB prevention, control, and management efforts, posing a substantial public health challenge. Despite the growing burden of NTM infections, comprehensive nationwide epidemiological surveys in China remain limited. There is an urgent need for detailed data on the dominant NTM species and their drug resistance profiles to optimize treatment regimens and support the development of an effective TB monitoring system (7). Such data are essential for informing public health policies and strategies aimed at controlling both NTM and TB infections. Therefore, this study systematically reviews and analyzes NTM reports published in mainland China and Taiwan between 2013 and 2024. Our objective is to provide a comprehensive understanding of the prevalence, distribution, and drug resistance patterns of NTM in China, thereby establishing a foundation for improved surveillance and management of NTM infections.

## 2 Method

### 2.1 Study design and ethical statement

This systematic review and meta-analysis adhered to the Preferred Reporting Items for Systematic Reviews and Meta-Analyses (PRISMA) guidelines to ensure methodological rigor. The protocol was prospectively designed and registered on the International Prospective Register of Systematic Reviews (PROSPERO; ID: CRD42024540102), promoting transparency. The study aimed to investigate the epidemiology, species distribution, and drug resistance of non-tuberculous mycobacteria (NTM) in China using data from publicly available literature. As no new human or animal subjects were involved, additional ethical approval or informed consent was not required.

### 2.2 Participants

The review included studies reporting clinical isolates from suspected tuberculosis (TB) patients in mainland China and Taiwan, collected between January 1, 2013, and December 31, 2024. Eligible participants were individuals of any age with NTM isolates confirmed by molecular sequencing, ensuring diagnostic accuracy and relevance to contemporary standards.

### 2.3 Systematic review protocol

The protocol was developed to systematically identify, select, and synthesize studies on NTM prevalence, species distribution, and drug resistance. The key objectives were: Estimating the pooled prevalence of NTM among suspected TB patients. Characterizing geographical and temporal trends in NTM isolation across China. Analyzing the species composition and drug resistance profiles of NTM isolates.

### 2.4 Search strategy

A comprehensive literature search was conducted to retrieve original research articles published between January 1, 2013, and December 31, 2024. The following databases were queried:

China National Knowledge Infrastructure (CNKI) and Wanfang: Searched using “non-tuberculous mycobacteria” and its Chinese synonyms to maximize coverage of studies from mainland China.

PubMed: Searched with the string: (“Nontuberculous Mycobacteria”[Mesh] OR NTM) AND (China [Affiliation]) AND (“2013/01/01”[Date-Publication]: “2024/12/31”[Date-Publication]) NOT (Review [Publication Type]) NOT (Meta-Analysis [Publication Type]).

Web of Science and The Cochrane Library: Applied the same search strategy as PubMed. A targeted search was also conducted to include all relevant NTM literature from Taiwan, ensuring geographical completeness.

### 2.5 Inclusion and exclusion criteria

To ensure a high-quality and relevant dataset, the following criteria were applied.

#### 2.5.1 Inclusion criteria

Studies must include NTM isolates collected between January 1, 2013, and December 31, 2024, reflecting current epidemiological trends. Only studies using molecular sequencing for NTM species identification were included, aligning with modern diagnostic standards. Studies must report detailed species-level data (e.g., number, types, and frequencies of NTM species). Only unique, non-duplicate clinical isolates from human specimens were considered to avoid bias.

#### 2.5.2 Exclusion criteria

Reviews, case reports, and meta-analyses were excluded due to their lack of original data.

Studies with NTM isolates from non-Chinese regions or non-clinical sources (e.g., environmental, animal, or laboratory-derived) were excluded. Multi-provincial studies lacking isolate counts per province were excluded to enable precise regional analysis.

Studies with vague or insufficient details on isolate sources (e.g., clinical setting, patient demographics, or specimen types) were excluded to ensure transparency. Studies focusing solely on specific NTM species without total clinical isolate numbers were excluded, as this context is critical for generalizability. These criteria ensured a robust dataset accurately representing NTM prevalence, diversity, and clinical impact in China from 2013 to 2024.

### 2.6 Data extraction and risk of bias assessment

For every included study, the following metadata were retrieved from the selected publications: first author, year when the study was published, study enrollment period, number of cases analyzed, research methods, sample origin and volume, NTM infection prevalence, and species distribution. Two researchers (X.X. and L.Y.) were responsible for both literature screening and data extraction, while a third investigator (Z.L.) conducted a review of their outcomes. Inconsistencies between the reviewers, whether in matters of study inclusion decisions or data extraction, were resolved through discussion to secure a consensus. Risk of bias was assessed using the Joanna Briggs Institute (JBI) Critical Appraisal Checklist for Prevalence Studies, which is suitable for observational studies like those included here (e.g., cross-sectional surveys on NTM prevalence). The checklist evaluates aspects such as sample representativeness, recruitment appropriateness, and statistical analysis. Assessments were performed independently by X.X., reviewed by L.Y., with disagreements resolved by Z.L.

### 2.7 Data analysis

Meta-analysis was conducted using Stata software to estimate the pooled prevalence of NTM and its 95% confidence interval (95% CI). Stratified analyses were subsequently performed to explore sources of heterogeneity, including geographical and temporal variations across China. Random-effects models were used for the meta-analysis, with full consideration of between-study heterogeneity. Egger’s test was applied to statistically assess publication bias (*p* < 0.05 was considered indicative of statistically significant publication bias). Publication bias was analyzed by multiple methods: Egger’s test for quantitative evaluation and funnel plots for qualitative detection, ensuring the reliability of findings.

## 3 Results

**Figure 1:**
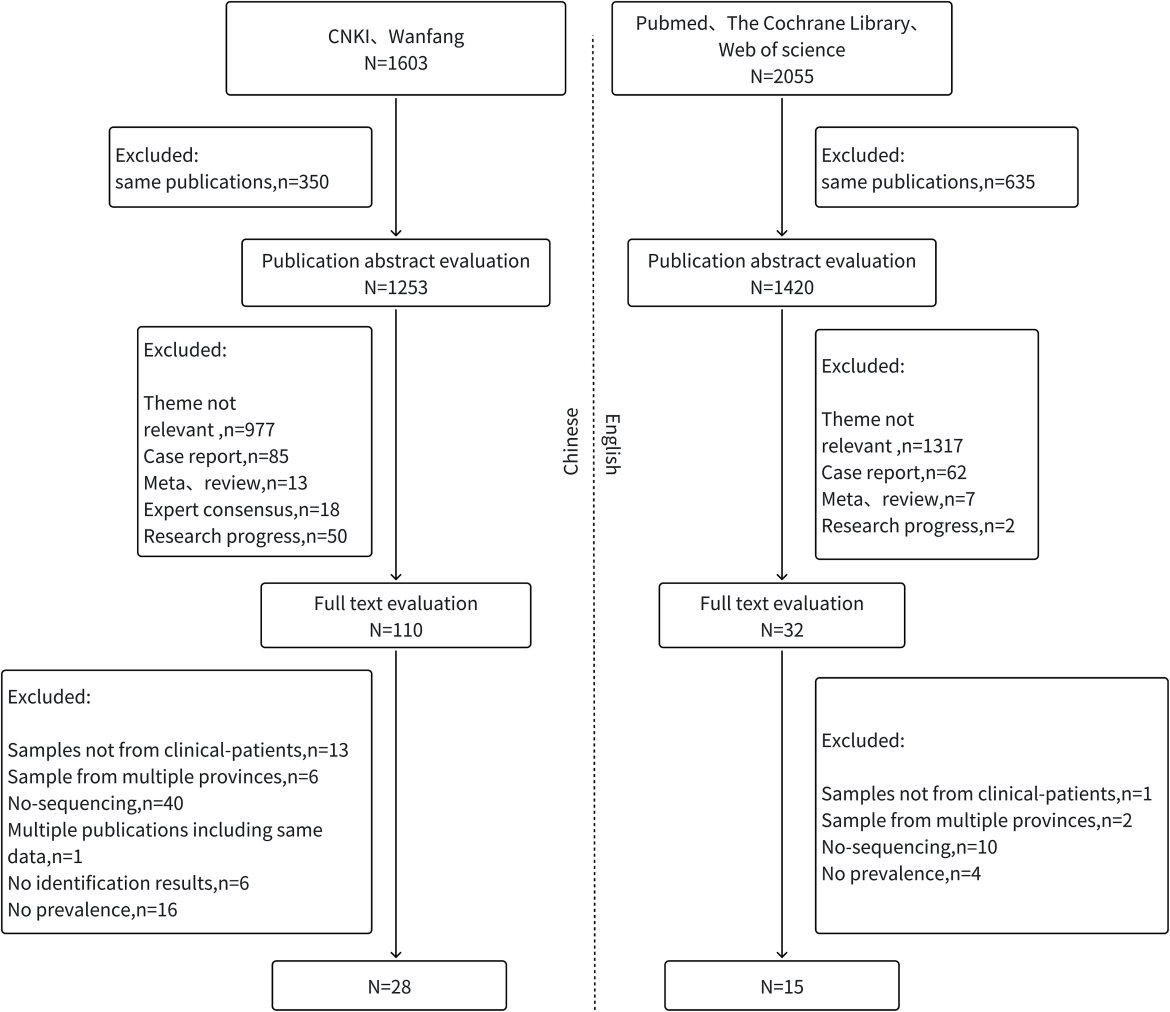
Flow diagram of study identification.

**Figure 2:**
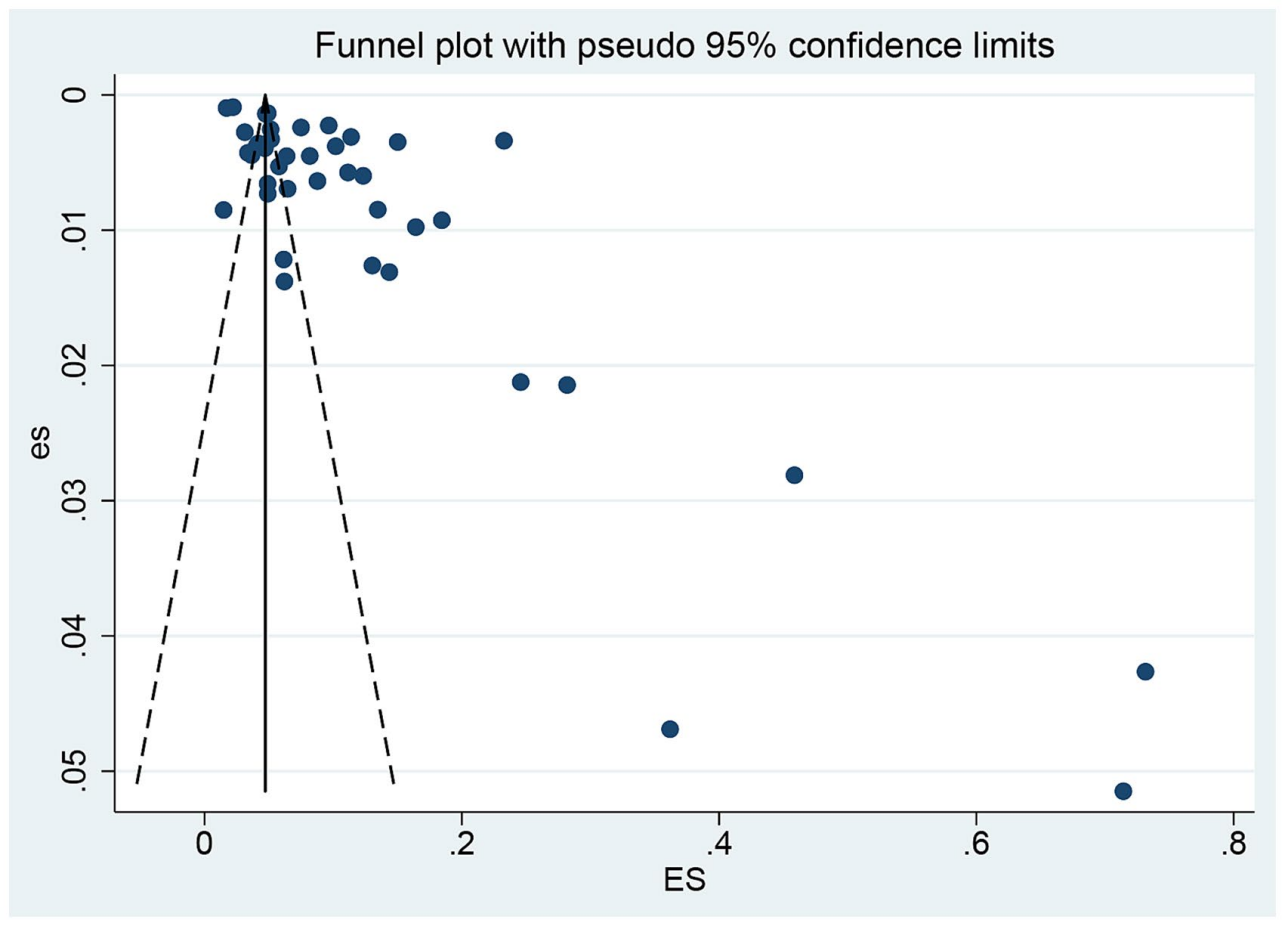
Funnel plot of the meta-analysis on the prevelance of NTM infections.

A systematic literature search across multiple databases identified 3,658 articles. After a rigorous multi-step screening process, 43 articles were included in the final analysis: 15 in English and 28 in Chinese, as shown in Figure 1. Publication bias was assessed using Egger’s weighted regression analysis, revealing significant bias (t = 4.69, *p* < 0.001), as depicted in Figure 2.

### 3.1 Prevalence of non-tuberculous mycobacteria (NTM)

#### 3.1.1 Overview of NTM isolation

From 2013 to 2024, the 43 included studies reported the isolation of 17,959 NTM isolates from clinical samples of 225,733 suspected tuberculosis (TB) patients across mainland China and Taiwan. Sequencing analysis identified 96 distinct NTM species, including 55 slowly growing mycobacteria (SGM) and 41 rapidly growing mycobacteria (RGM), excluding species with undetermined subtypes. The four most frequently isolated species, detailed in Table 1, were *M. intracellulare*, *M. avium complex (MAC)*, *M. abscessus*, and *M. avium*.

**Table 1:** The distribution and NTM survey of the ten most common strains in each province.

NTM Species	Guangdong	Zhejiang	Shanghai	Anhui	Hunan	Shandong	Fujian	Sichuan	Taiwan	Hainan	Beijing	Hubei	Gansu	Jilin	Henan	Jiangsu	Jiangxi	Shanxi
*M. intracellulare*	61	1,521	282	1,485	415	857	302	182	90	135	175	189	67	30	49	41	10	0
*MAC*	1,305	0	447	0	0	0	191	0	94	0	67	0	1	10	0	0	0	0
*M. abscessus*	96	236	330	254	383	0	142	0	148	102	7	68	0	0	3	0	6	0
*M. avium*	16	271	82	56	247	27	138	183	39	6	74	9	0	0	2	0	0	2
*M. kansasii*	302	221	112	140	31	136	19	43	36	2	34	16	11	10	5	4	0	1
*MKC*	0	0	693	0	0	0	0	0	0	0	0	0	1	0	0	0	0	0
*M. gordonae*	137	71	23	13	258	22	53	14	38	8	0	25	0	16	0	0	2	0
*M. fortuitum*	290	33	25	10	39	47	23	26	32	14	15	24	0	5	0	0	0	0
*M. chelonae-abscessus*	0	182	0	0	0	124	0	193	0	0	0	0	0	0	0	0	0	0
*MABC*	0	0	268	0	0	0	0	0	0	0	0	0	1	0	0	0	0	0
NTM total	3,871	2,745	2,707	1988	1,525	1,258	959	712	529	491	467	385	96	79	65	60	19	3
Number of subspecies	26	47	36	15	16	6	30	10	24	50	19	16	9	5	5	2	4	2

The first column was sorted in descending order based on the total detection counts of each NTM subspecies across all provinces, with *Mycobacterium intracellulare* showing the highest overall count. The first row was sorted in descending order on the total detection counts of NTM in each province, with Guangdong Province exhibiting the highest prevalence. The shaded part represents the bacterial strain with the largest number of identifications in this province. References associated with the data presented in this table are elaborated on in Supplementary Table S8.

#### 3.1.2 Geographic distribution and species variation

The dominant NTM species varied by province. *M. intracellulare* was predominant in most regions, while *M. kansasii complex (MKC)* led in Shanghai, *MAC* in Guangdong, and *M. chelonae-abscessus complex* in Sichuan. The provinces reporting the highest number of NTM isolates were Guangdong (3,871 strains, 26 species), Zhejiang (2,745 strains, 47 species), Shanghai (2,707 strains, 36 species), and Anhui (1,988 strains, 15 species). Hainan Province exhibited the greatest species diversity, with 50 identified NTM species (Table 1).

#### 3.1.3 Temporal trends

Seventeen studies provided annual isolation data (Table 2), indicating a peak in NTM isolation between 2018 and 2021 (>1,400 strains per year), followed by a decline in 2022 and 2023 (<750 strains per year). Despite this recent decrease, an overall upward trend in NTM isolation has been observed since 2009.

**Table 2:** NTM isolation in several provinces during the period from 2009 to 2023.

Year	Guangdong	Anhui	Hunan	Sichuan	Hainan	Beijing	Taiwan	Zhejiang	Hubei	Shanghai	Jiangsu	Total
2009						59						59
2010												0
2011												0
2012								93				93
2013						71	529					600
2014					5			86				91
2015					89			72				161
2016			336		22			80	54			492
2017			360	11	63			0	69			503
2018	901		388	37	79			51	97		60	1,613
2019	1,184		441	110	72	579		54	156	7		2,603
2020	1,078			102	87			69	123	14		1,473
2021	1,173	547		124	68	617		86		34		2,649
2022		521		203								724
2023		521										521
Total	4,336	1,589	1,525	587	485	1,326	529	591	499	55	60	11,582

Of the included literature, only 17 studies provided detailed annual counts of identified NTM isolates. The temporal range of NTM sources spans from 2009 to 2023. References associated with the data presented in this table are elaborated on in Supplementary Table S8.

#### 3.1.4 Pooled prevalence

The pooled prevalence of NTM infection among suspected TB patients in mainland China was 11.27% (95% CI: 9.89–12.65%; n/N = 17,430/224,783), as presented in Table 3.

**Table 3:** NTM isolation rates in mainland China and Taiwan area.

Subgroup	Classification	No. of study	Prevalence of NTM (95%CI)	Proportion of NTM in Mycobacterium (%)	Heterogenity, *I^2^*	Heterogenity *p* value	Egger’s test, *t*	Egger’s test, *p* value
Overall effect of mainland China		42	11.272 [9.890, 12.654]	17,430/224783	99.54%	0.000	4.69	0.000
Region	South-east	24	12.825 [10.819, 14.830]	12,091/146033	99.62%	0.000	3.59	0.002
	Central	9	7.388 [4.424, 10.351]	3985/40102	99.15%	0.000	−0.27	0.798
	North-east	5	24.182 [16.424, 31.941]	546/6191	98.98%	0.000	3.86	0.031
	South-west	2	2.303 [2.135, 2.472]	712/30501	100%	0.000	-	-
	North-west	2	4.908 [3.951, 5.865]	96/1956	0	0.000	-	-
	Taiwan	1	55.684 [52.459, 58.874]	529/950	-	-	-	-
	NTM < 100	12	14.828 [11.381, 18.275]	613/8671	98.01%	0.000	4.92	0.001
	200 > NTM > =100	12	11.027 [8.939, 13.116]	1559/24228	98.08%	0.000	14.66	0.000
	NTM > =200	19	12.208 [10.015, 14.401]	15,787/192834	99.79%	0.000	5.39	0.000

Provinces locate in cental region: Anhui, Henan, Hubei, Hunan, Shanxi, Jiangxi; Provinces locate in south-east region: Fujian, Guangdong, Hainan, Jiangsu, Shandong, Shanghai, Zhejiang; Provinces locate in south-west region: Sichuan; Provinces locate in north-west region: Gansu; Provinces locate in north-east region: Beijing, Jilin; References associated with the data presented in this table are elaborated Supplementary Table S8.

#### 3.1.5 Geographic variation in prevalence

**Figure 3:**
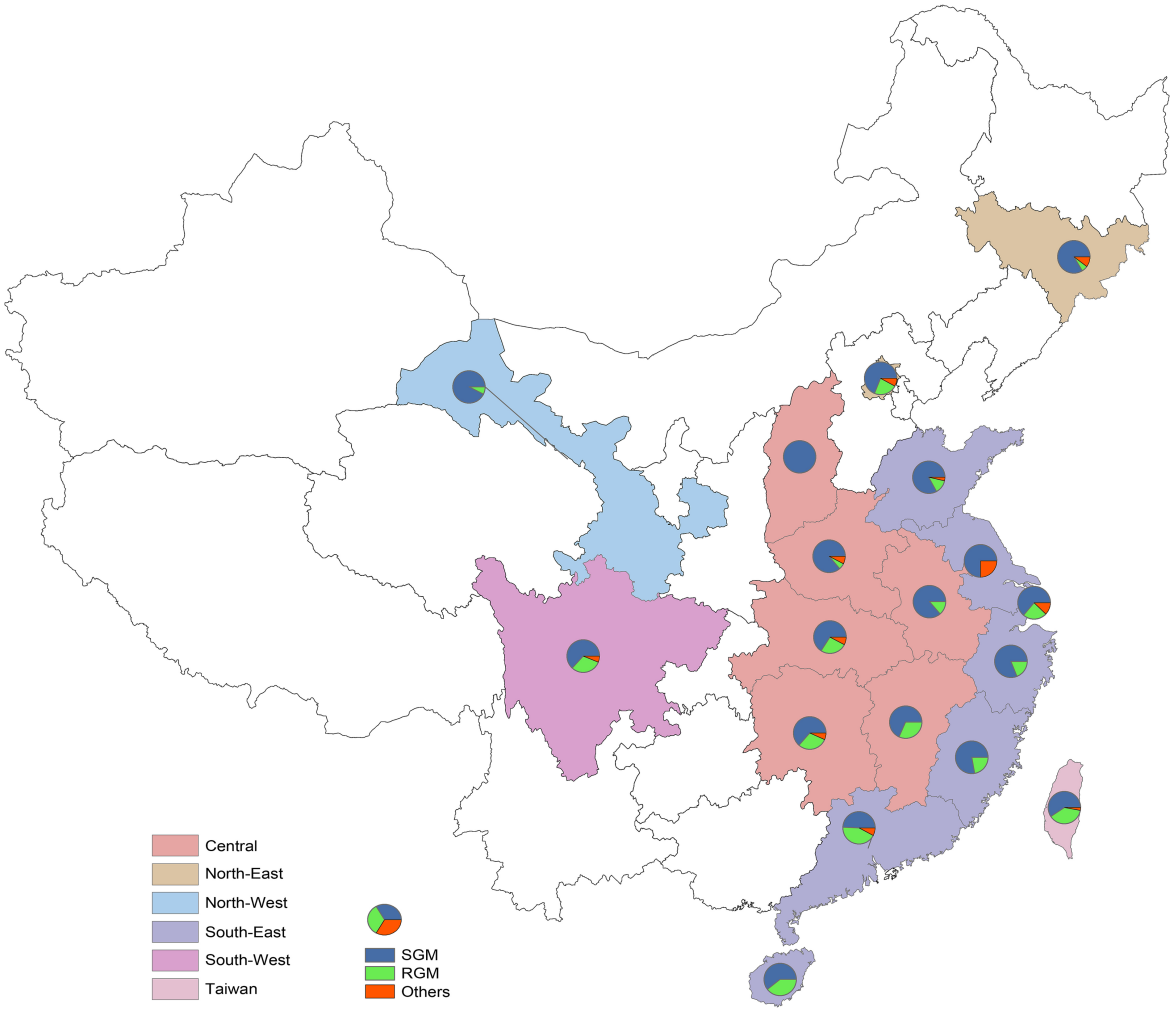
The prevalence of NTM infections in individual province in mainland China and Taiwan. RGM, rapid growing mycobacteria, SGM, slow growing mycobacteria.

NTM prevalence varied significantly across regions, with higher rates in the northeast compared to the southwest and in coastal areas compared to inland regions. The southeast region reported a prevalence of 12.83% (95% CI: 10.82–14.83%), exceeding the inland southwest region’s 2.30% (95% CI: 2.14–2.47%). Similarly, the northeast region’s prevalence of 24.18% (95% CI: 16.42–31.94%) surpassed the Northwest’s 4.91% (95% CI: 3.95–5.87%). Provinces with notably high prevalence included Jilin (73.15%, *p* < 0.001), Shanghai (19.21%, *p* < 0.001), Guangdong (27.38%, *p* < 0.001), and Fujian (14.08%, *p* < 0.001). NTM isolation patterns and the proportions of RGM and SGM by province are illustrated in Figure 3.

#### 3.1.6 SGM and RGM distribution

SGM accounted for 68.07% (12,225/17,959) of isolates, while RGM comprised 26.57% (4,771/17,959), reflecting a broader distribution of SGM. Table 4 lists the most prevalent SGM and RGM species (based on studies with >2 reports). Among SGM, the leading species were *MAC* (8.27, 95% CI: 5.16–11.38%), *M. intracellulare* (4.03, 95% CI: 3.42–4.64%), *M. avium* (0.60, 95% CI: 0.48–0.73%), and *M. kansasii* (0.57, 95% CI: 0.46–0.68%). For RGM, the primary species included *M. chelonae-abscessus* (2.14, 95% CI: 1.15–3.13%), *M. abscessus* (1.90, 95% CI: 1.55–2.25%), *M. massiliense* (0.31, 95% CI: 0.15–0.46%), and *M. fortuitum* (0.22, 95% CI: 0.16–0.27%). Isolation details for other NTM species (reported in ≤2 studies) are provided in Supplementary Table S1.

**Table 4:** Species distribution among the NTM isolates from mainland China.

Classification	NTM species	No. of studies	Prevalence of NTM (95% CI)	n/N	Heterogenity, *I^2^*	Heterogenity *P* value	Egger’s test, *t*	Egger’s test, *P* value
SGM	*M. kansasii*	39	0.570 [0.464, 0.677]	1166/205364	95.622%	0.000	5.89	0.000
	*M. intracellulare*	37	4.030 [3.419, 4.641]	5898/189426	98.815%	0.000	5.28	0.000
	*M. avium*	34	0.603 [0.481, 0.725]	1121/186718	95.443%	0.000	4.93	0.000
	*M. gordonae*	29	0.334 [0.258, 0.410]	692/190540	94.874%	0.000	5.25	0.000
	*M. scrofulaceum*	15	0.035 [0.019, 0.051]	56/120827	41.872%	0.045	4.26	0.001
	*M. xenopi*	10	0.031 [0.012, 0.050]	32/79543	49.364%	0.038	4.76	0.001
	*M. colombiense*	11	0.048 [0.018, 0.078]	45/63757	64.844%	0.002	7.26	0.000
	*M. lentiflavum*	11	0.052 [0.036, 0.068]	65/108904	20.083%	0.252	2.97	0.016
	*M. marseillense*	7	0.173 [0.075, 0.271]	58/27755	75.516%	0.000	2.39	0.062
	*M. simiae*	5	0.238 [0.090, 0.386]	51/17706	91.509%	0.000	2.83	0.066
	*M. szulgai*	8	0.035 [0.015, 0.056]	23/46015	17.580%	0.291	2.98	0.025
	*M. malmoense*	5	0.032 [0.010, 0.054]	16/36972	20.859%	0.282	1.65	0.197
	*M. terrae*	6	0.017 [0.006, 0.029]	13/53264	0.000%	0.587	3.23	0.032
	*M. neoaurum*	6	0.022[−0.001, 0.045]	10/26678	18.030%	0.29663	5.52	0.005
	*M. marinum*	6	0.141[−0.020, 0.303]	80/38929	94.181%	0.000	3.43	0.026
	*M. triplex*	4	0.022 [0.004, 0.041]	8/25960	0.000%	0.531	4.08	0.055
	*M. seoulense*	4	0.010 [0.000, 0.019]	6/42199	0.000%	0.598	4.76	0.041
	*M. shimoidei*	4	0.078 [0.015, 0.141]	8/7551	0.000%	0.547	2.32	0.146
	*MAC*	6	8.267 [5.155, 11.379]	2047/37086	99.190%	0.000	1.27	0.273
	*M. asiaticum*	5	0.023 [0.002, 0.043]	7/21218	0.000%	0.697	6.74	0.007
	*M. interjectum*	4	0.008 [−0.004, 0.019]	6/33857	16.772%	0.307	5.33	0.033
	*M. mantenii*	3	0.012 [−0.011, 0.036]	5/19298	33.105%	0.224	5.78	0.109
	*M. arupense*	3	0.113 [−0.155, 0.381]	4/23863	32.190%	0.228	5.75	0.110
	*M. paragordonae*	5	0.023 [−0.005, 0.051]	15/36084	57.612%	0.051	3.50	0.040
	*M. parascrofulaceum*	4	0.038 [−0.001, 0.078]	19/37616	69.532%	0.199	3.11	0.090
	*M. timonense*	3	0.059 [−0.016, 0.133]	9/17891	59.126%	0.087	3.89	0.160
RGM	*M. fortuitum*	35	0.215 [0.159, 0.271]	597/200211	93.081%	0.000	4.97	0.000
	*M. abscessus*	33	1.901 [1.550, 2.252]	1844/110840	97.338%	0.000	6.10	0.000
	*M. chelonae*	12	0.055 [0.023, 0.087]	63/77441	78.141%	0.000	4.74	0.001
	*M. smegmatis*	8	0.013 [0.004, 0.022]	12/57217	0.000%	0.698	10.80	0.000
	*M. phlei*	6	0.007 [−0.000, 0.015]	8/49314	0.000%	0.487	8.63	0.001
	*M. massiliense*	8	0.307 [0.152, 0.462]	110/36441	91.754%	0.000	3.68	0.010
	*M. monacense*	5	0.011 [0.001, 0.021]	7/42610	0.000%	0.693	10.06	0.002
	*M. chelonae-abscessus*	5	2.136 [1.147, 3.125]	1739/94823	99.655%	0.000	2.27	0.108
	*M. senegalense*	6	0.143 [0.021, 0.266]	15/8453	42.648%	0.121	4.94	0.008
	*M. mucogenicum*	4	0.037 [−0.049, 0.123]	12/28588	71.869%	0.014	3.13	0.089
	*M. septicum*	3	0.037 [−0.005, 0.078]	8/20129	36.027%	0.209	1.72	0.336
	*M. phocaicum*	5	0.053 [0.001, 0.105]	8/7580	0.265%	0.405	8.73	0.003
	*M. aubagnense*	3	0.042 [−0.016, 0.100]	3/4734	0.000%	0.596	709.72	0.001
	*M. peregrinum*	8	0.020 [0.004, 0.035]	20/61057	26.535%	0.217	5.01	0.002
	*M. wolinskyi*	4	0.007 [−0.002, 0.016]	4/33122	0.000%	0.635	648.11	0.000
	*M. porcinum*	4	0.0035 [0.009, 0.061]	7/19642	0.000%	0.983	1.55	0.262

### 3.2 Non-mycobacterial contaminants

A total of 135 non-mycobacterial contaminants were identified across the included studies (Supplementary Table S2). The most frequently isolated genus was *Nocardia* accounting for 55 isolates (40.74%), with *N. farcinica* being the dominant species at 19 isolates (14.07%). Next was *Gordonia,* with 34 isolates (25.19%). Followed by *Tsukamurella* with 10 isolates (7.41%). Other contaminants included *Corynebacterium* and *Streptomyces* (2 isolates each, 1.48% respectively), single isolates of *Actinomadura* and *Halomonas* (0.74% each), and 30 isolates categorized as “Others” (22.22%). The diversity underscores the presence of environmental saprophytes in clinical samples, emphasizing the need for accurate microbiological differentiation.

### 3.3 Comorbidities

Data from five studies on comorbidities associated with non-tuberculous mycobacterial (NTM) infections are summarized in Supplementary Table S3. The most common comorbidities were: HIV: 95 cases, the most frequent comorbidity. Bronchiectasis: 74 cases. Chronic obstructive pulmonary disease (COPD): 23 cases. Diabetes: 38 cases. Hypertension: 21 cases. Hepatitis B: 15 cases, alongside other liver diseases. These findings highlight the clinical complexity of NTM infections, which frequently occur in patients with underlying pulmonary and metabolic conditions.

### 3.4 Culture and identification methods for NTM

Over the past decade in China, NTM isolation and identification have primarily relied on Lowenstein-Jensen (L-J) medium, p-nitrobenzoic acid (PNB), thiophene-2-carboxylic acid hydrazide (TCH), and BACTEC^™^ culture systems (Supplementary Table S4). Preliminary identification commonly utilized the MPB64 immunochromatographic assay, while polymerase chain reaction (PCR) and sequencing of conserved genes (e.g., 16S rRNA, hsp65, rpoB) were essential for species and subtype determination (Supplementary Table S5). Recently, matrix-assisted laser desorption/ionization-time of flight mass spectrometry (MALDI-TOF MS, Vitek MS v3.0) has emerged as a rapid, cost-effective alternative to traditional DNA sequencing, reducing turnaround time and labor intensity. All included studies employed both multilocus and single-gene sequencing, with 16S rRNA, hsp65, and rpoB being the most commonly targeted genes, supplemented occasionally by ITS and Rv0557. Advances in chip technology, such as Mycobacterium gene chip kits using fluorescence probes and melting curve methods, have also gained popularity due to their efficiency.

### 3.5 Drug susceptibility testing and resistance rates of NTM

Of the 43 studies, 16 reported drug susceptibility testing (DST) results for NTM, utilizing five different methods (Supplementary Table S6). The most common methods were microbroth dilution and traditional proportion methods. A total of 34 drugs were tested against 18 NTM species and 10 NTM complexes (Supplementary Table S7). Among the most prevalent clinical isolates, *M. intracellulare* and *M. abscessus* exhibited the highest resistance rates, often exceeding 90% for first-line anti-tuberculosis (TB) drugs, followed by *M. kansasii* and *M. scrofulaceum*. While most NTM species demonstrated resistance to first-line TB drugs, linezolid (LZD), clofazimine (CFM), amikacin (AMK), tobramycin (TOB), and clarithromycin (CLA) showed efficacy, suggesting their potential as preferred therapeutic options for NTM infections.

## 4 Discussion

### 4.1 Overall prevalence and temporal trends in NTM isolation

Globally, NTM prevalence varies significantly. Turkey reported a prevalence of 7.54% (n/N = 1,304/17,293) from 2012–2022 (8), while Africa’s prevalence ranged from 0.2 to 28% between 2000 and 2021 (9). In Europe, NTM pulmonary disease incidence is relatively low, with rates per 100,000 people varying by region: UK (0.9–7.0), France (1.3–13.6), Germany (3.9–8.2), Italy (3.8–10.4), and Spain (3.3–8.4) (10). North America reports increasing prevalence, with U.S. incidence rates of 1.4–47.5 per 100,000 and similar trends in Canada (11). China’s NTM prevalence (11.27%) exceeds many of these estimates, reflecting a significant public health burden. Unlike Denmark, which centralizes mycobacterial diagnosis at the International Reference Laboratory for Mycobacteriology (IRLM) with stringent standards and high positive predictive value (12), China lacks a mandatory NTM reporting system. Detection is primarily regional, with economically developed areas like Shanghai and Shenzhen leading through cohort studies, but nationwide standardization remains limited, resulting in fragmented and heterogeneous data.

Despite the escalating global burden of NTM infections, China lacks comprehensive nationwide epidemiological studies. Over the past two decades, individual investigations have reported widely varying NTM prevalence rates, underscoring the heterogeneity of these organisms. Historical tuberculosis surveys illustrate this trend, with NTM isolation rates rising from 4.3% in 1979 to 11.1% in 2000, and reaching 22.9% by 2010 (13). Meta-analyses further highlight this variability: Yu et al. estimated a prevalence of 6.3% (95% CI: 5.4–7.4%) based on studies before July 31, 2015 (6), while Zhou et al. reported 4.66% (95% CI: 4.58–4.74%) for 2000–2019 (5). With new NTM-related literature emerging and increased attention on NTM epidemiology in mainland China and Taiwan, our study synthesized data from January 1, 2013 to December 31, 2024. Our estimated prevalence exceeds prior meta-analyses, confirming an upward trend in China. This finding emphasizes the urgent need for improved surveillance and management strategies.

Analysis of NTM isolation trends from 17 studies (2009–2023) revealed a pronounced nationwide increase accompanied by significant geographic heterogeneity. Initial surveillance in 2009 documented merely 59 isolates, contrasting sharply with the substantial rise by 2023, reflecting enhanced diagnostic capabilities and growing clinical recognition. Interprovincial disparities were evident: Zhejiang exhibited an early peak followed by decline, likely indicative of effective local control measures, whereas Sichuan, Hunan, Hainan, Hubei, and Shanghai demonstrated sustained yet divergent upward trends. Temporal anomalies, including reductions in Hubei (2020) (14) and a transient dip in Sichuan (15, 16) post-2021, coincided with the COVID-19 pandemic, suggesting disruptions in healthcare access influenced NTM detection. These patterns underscore a complex NTM epidemiology driven by multifactorial determinants, including advancements in detection methodologies, environmental factors, and evolving public health priorities.

### 4.2 Geographical variations and species distribution

The distribution of NTM across China reveals significant geographical heterogeneity, influenced by environmental and socioeconomic factors. Coastal provinces, such as Hainan and Zhejiang, exhibit greater NTM diversity than inland regions, likely due to humid subtropical climates and advanced diagnostic infrastructure in economically developed areas (Figure 3). Species distribution also varies regionally: *M. intracellulare* predominates in Fujian and Jilin, while *M. abscessus* is more common in Guangdong and Taiwan (17). This reflects latitude-dependent patterns, with warmer regions favoring RGM like *M. abscessus* due to their thermotolerance and biofilm-forming abilities (18, 19). The southeastern coast, with higher surface water exposure and urbanization, shows elevated RGM prevalence (20). However, Jilin’s notably high prevalence (73.1%) stems from a methodological bias: a single-center study (21) using DNA microarrays (75.9% detection rate) in immunocompromised TB patients, which likely inflates the true rate. These disparities underscore the need for multi-center studies with standardized methods, such as targeted next-generation sequencing (tNGS), to distinguish genuine biogeographical trends from detection artifacts.

Consistent with previous reviews by Yu et al. (6) and Zhou et al. (5), our analysis of the 43 included studies revealed that SGM significantly outnumbered RGM. Among SGM, the *MAC* and *MKC* were dominant. *MAC*, encompassing over 20 species with *M. intracellulare* and *M. avium* as the most prevalent, is a leading cause of pulmonary NTM disease globally. Its ability to evade neutrophil killing, as demonstrated by Nakamura et al., involves inducing neutrophil extracellular trap (NET) formation and interleukin-8 (IL-8) release, triggering neutrophil aggregation and amplifying inflammation through NET-dependent secretion of matrix metalloproteinase-8 (MMP-8) and -9 (MMP-9), exacerbating pulmonary infection (22). The non-specific clinical and pathological features of *MAC* infections, which mimic tuberculosis, pose significant diagnostic and therapeutic challenges (15). For *MKC*, *M. kansasii* was the most prevalent subtype, particularly among HIV-infected patients (23), aligning with Yu et al.’s findings (6). Among RGM, the *MABC* was predominant, comprising *M. abscessus subsp. abscessus*, *M. abscessus subsp. massiliense*, and *M. abscessus subsp. bolletii*. *MABC* is notorious for its treatment resistance (24), driven by synergistic interactions among immune responses, virulence traits, and genomic features, including: activation of Type I/II interferon pathways and TLR2-mediated TNF-*α* production; distinct morphotypes, with rough variants (exhibiting cording morphology due to mmpL4b deletion and glycopeptidolipid deficiency) evading immune responses and smooth variants suppressing IFN-I signaling and phagolysosomal fusion; subspecies differentiation via housekeeping gene sequencing (19); *MABC*-exclusive genes and specialized plasmids (e.g., mercury resistance plasmids) enhancing respiratory tract colonization (25). Our findings confirm Yu et al.’s observation that the *M. chelonae-abscessus complex* is the predominant taxonomic cluster within RGM, reinforcing its critical role in NTM epidemiology (6).

### 4.3 Novel and rare NTM species

In addition to common NTM species, our review identified rare subtypes first reported in China, including *M. saskatchewanense* (26), *M. rutilum* (27), and *M. paraense* (28) from various provinces. Hainan and Taiwan exhibited exceptional species diversity, isolating multiple rare subspecies such as *M. nebraskense*, *M. algericus*, *M. sherrisii*, *M. sinense*, *M. conceptionense*, *M. eburneum*, *M. frederiksbergense*, *M. brisbanense*, *M. cosmeticum*, *M. llutzerense*, *M. canariasense*, *M. moriokaense*, and *M. longobardum* (17). Notably, *M. nebraskense*, a rare slowly growing NTM first isolated in 2004 at the University of Nebraska (29), is infrequently reported in human infections. Rajani et al. suggests asymptomatic infections are more common in females (30), with Metersky et al. reporting 11 cases (8 females, 3 males) (31). *M. frederiksbergense*, a rapidly growing scotochromogenic mycobacterium identified in 2001 from coal tar-contaminated soil in Denmark (32), is also rare in China. The high incidence of these rare strains in subtropical/tropical regions like Hainan and Taiwan likely reflects environmental factors, such as climate and geography, combined with lifestyle and behavioral patterns, highlighting the need for region-specific surveillance.

### 4.4 Drug susceptibility testing and resistance profiles

Among the five DST methods reported across the 16 studies, the traditional proportion method, endorsed by the World Health Organization (WHO) as the gold standard for tuberculosis drug sensitivity testing (33), maintained consistent use (1–2 publications annually from 2015–2022). Despite its reliability, its 4-week testing cycle, complex procedures, and high costs (33) limit its utility for rapid NTM diagnosis. In contrast, the broth microdilution method, recommended by the Clinical and Laboratory Standards Institute (CLSI), gained prominence with 7 publications from 2021–2024, offering advantages in quantifying antimicrobial efficacy via minimum inhibitory concentration (MIC) values (34). The MicroDST^™^ microplate method emerged as the most frequently cited system in recent years, attributed to its operational simplicity (60% reduction in testing time), cost-effectiveness (40% lower reagent costs), and standardization (35). Conversely, the absolute concentration method (reported only in 2018) and Alamar-Blue method (single report) have declined in use due to challenges such as strict inoculum control and subjective result interpretation (36, 37). The 16 studies revealed significant heterogeneity in NTM resistance to 34 antimicrobial agents (Supplementary Table S7). For *Mycobacterium abscessus* and *M. intracellulare*-the most clinically challenging species-*M. abscessus* exhibited resistance rates exceeding 94% to isoniazid (INH) and streptomycin (STR) but showed low resistance (<7%) to tigecycline (TGC), clarithromycin (CLA), and cefoxitin (FOX). *M. intracellulare* displayed >90% resistance to INH, yet retained 77% sensitivity to CLA. These patterns are driven by NTM-specific multidrug resistance mechanisms, including: (1) a lipid-rich cell wall barrier impeding drug penetration; (2) drug target mutations (e.g., RRL gene mutations causing macrolide resistance); and (3) overexpression of efflux pumps, particularly for fluoroquinolones and tetracyclines (38). Notably, RGM showed universal resistance to *β*-lactams like imipenem (IPM) and moxifloxacin (MOX) but 100% sensitivity to TGC, which penetrates biofilms by inhibiting the ribosomal 30S subunit (39, 40), offering a promising option for multidrug-resistant RGM infections.

Significant variations in resistance rates were observed across testing methods for the same species and drug (Supplementary Table S7). For example, *M. abscessus* resistance to ethambutol (EMB) ranged from 100% (proportion method) to 0% (broth dilution), while *M. intracellulare* resistance to minocycline (MIN) differed by 91.83 percentage points between MicroDST^™^ and broth dilution methods. These discrepancies stem from: methodological differences (e.g., colony-forming unit counting in the proportion method versus liquid culture in dilution methods); inconsistent critical concentration settings, especially for NTM with undefined breakpoints; strain subtype heterogeneity (e.g., erm gene expression differences between *M. abscessus subsp. massiliense* and *M. abscessus subsp. abscessus*) (5). Clinicians should prioritize multicenter data based on locally prevalent strains and integrate molecular testing (e.g., target gene sequencing) to guide treatment decisions when DST data are limited.

Among first-line anti-tuberculosis drugs, only EMB retained partial sensitivity to NTM (54.1%), while second-line drugs like AMK, CLA, and LZD demonstrated broad-spectrum activity (sensitivity >50%), aligning with Zhou et al.’s proposed NTM ladder treatment protocol (5). Kadota et al. reported that triple regimens combining macrolides and aminoglycosides increased remission rates for *MAC* pulmonary disease by 35% (41), likely due to enhanced drug penetration and complementary mechanisms (42). Novel agents, such as amikacin liposome inhalation suspension (in phase I/II trials for *MAC* pulmonary disease in the U.S.) (43, 44) and omadacycline (82% *in vitro* bacteriostatic rate against *M. abscessus complex*) (45–47), show promise. Future efforts should accelerate the clinical translation of NTM-specific drugs and develop individualized combination regimens guided by molecular DST monitoring.

### 4.5 Non-mycobacterial contaminants

Our review identified 135 non-mycobacterial contaminants, predominantly *Nocardia* (40.74%, mainly *N. farcinica*) and Gordonia (25.19%, including *G. aichiensis* and *G. otitidis*). *Nocardia* is ubiquitous in the environment and can cause skin, pulmonary, or brain infections, particularly in immunocompromised patients, though one-third of cases occur in immunocompetent individuals with chronic lung diseases or bronchiectasis (48, 49). Wang et al. noted *N. farcinica* and *N. cyriacigeorgica* as the most common *Nocardia* species in mainland China (50). *Gordonia* species, initially considered opportunistic pathogens, are gaining attention for their ability to biodegrade environmental pollutants and synthesize beneficial organic compounds (51). Immunocompromised patients (e.g., those with HIV, hypogammaglobulinemia, leukemia, or chronic hepatitis B) or those with underlying conditions like diabetes or COPD are particularly susceptible to *Gordonia* infections (52). Other contaminants, such as *Streptomyces tubbatahanensis* (first identified in Hainan) (53), *Tsukamurella* (54), and *Halomonas* (with some pathogenic strains) (55, 56), highlight the complex diversity of environmental microorganisms in clinical samples. These findings emphasize the importance of optimizing sample collection standards and enhancing environmental microorganism reference databases.

### 4.6 Comorbidities and clinical implications

NTM infections predominantly affect individuals with underlying conditions, such as immunocompromised HIV patients (57), or those with cystic fibrosis, chronic lung diseases, or bronchiectasis (58–60). Our review identified HIV as the most common comorbidity (95 cases), with Miao et al. noting *MAC* as the predominant NTM species in HIV patients (61). Bronchiectasis (74 cases) and COPD (23 cases) were significant pulmonary comorbidities, with *M. intracellulare* and *M. abscessus* identified as primary pathogens in Hubei (14), consistent with Lou’s et al. (62) findings of higher bronchiectasis incidence in patients with these species. Metabolic conditions like diabetes (38 cases), hypertension (21 cases), and hepatitis B (15 cases) were also prevalent (15, 63). Although our review focused on pulmonary infections, extrapulmonary NTM infections, such as bone and joint diseases (e.g., tenosynovitis, arthritis, vertebral osteomyelitis) caused by *M. kansasii* and *M. marinum* (64, 65), and skin and eye infections caused by *MAC* and *M. chelonae* (66, 67), are notable in immunocompromised populations. These findings underscore the broad clinical impact of NTM and the need for heightened vigilance in patients with underlying conditions, particularly HIV and bronchiectasis. Future research should prioritize extrapulmonary infections and elucidate pathogenic mechanisms to inform targeted prevention and treatment strategies.

### 4.7 Advances in NTM identification

NTM identification in China has evolved significantly from 2013 to 2024, as detailed in Supplementary Table S5. Molecular techniques, particularly PCR-based amplification and sequencing of 16S rRNA, hsp65, and rpoB genes, dominate, with 16S rRNA sequencing cited in 31 publications. These methods enable precise subtype identification, reflecting their reliability. Commercial kits, such as the GenoType Mycobacterium CM/AS Kit (68), Mycobacteria Identification Array Kit (CapitalBio), and MeltPro^®^ Mycobacteria Identification Kit (1), have gained prominence for their sensitivity and rapid turnaround, with 14 publications citing the CapitalBio kit (2015–2024). Immunochromatography (e.g., MPB64-based kits) supports preliminary identification but requires sequencing confirmation due to potential false negatives. Emerging technologies like DNA microarrays and whole-genome sequencing (WGS), reported increasingly in 2021–2024, offer high accuracy but are limited by cost and technical demands (69). Matrix-assisted laser desorption/ionization time-of-flight mass spectrometry (MALDI-TOF MS) is also gaining traction for proteomic-based identification (70). Despite these advances, limitations persist: commercial kits may have restricted probe sets, and advanced methods like WGS require specialized equipment, hindering adoption in resource-limited settings.

### 4.8 Temporal and regional insights

Compared to Zhou et al.’s 2000–2019 analysis (5), our study (2013–2024) identified a “decline followed by rise” pattern in NTM isolation rates, likely influenced by disruptions in tuberculosis detection during COVID-19 pandemic control measures. For the first time, our review included data from Taiwan and Hainan, revealing cross-border transmission patterns in unique marine and subtropical environments, suggesting water bodies as potential transmission media. These findings underscore the importance of integrating regional ecological factors into NTM surveillance strategies.

### 4.9 Limitations

Our findings, which underscore the growing burden of non-tuberculous mycobacteria (NTM) infections in China driven by environmental, clinical, and diagnostic factors, are constrained by limitations that affect result robustness and generalizability. First, methodological inconsistencies across studies compromise NTM prevalence estimate reliability and cross-study comparability: for instance, Jilin’s elevated prevalence reflects bias from a single-center study using a highly sensitive detection method (skewing results relative to multi-site data), while variability in diagnostic tools, study populations, and reporting practices further hinders direct comparisons. Second, data primarily sourced from large tertiary hospitals introduces urban-centric bias, failing to capture NTM infections in rural/remote areas with limited access to advanced diagnostics and potentially underestimating the true burden in under-resourced regions. Third, the limited number and small sample size of included studies from certain regions (e.g., Taiwan) may restrict the generalizability of region-specific findings. Finally, the absence of longitudinal cohort studies limits validation of dynamic NTM drug resistance trends; current cross-sectional data are insufficient to confirm resistance profile changes over time—critical for guiding treatment guideline updates. Finally, reliance on published literature introduces publication bias (Egger’s test, *p* < 0.001), where studies with “significant” findings (e.g., high prevalence, rare species identification) are overrepresented, while non-significant/negative results are underreported, further skewing prevalence estimates.

### 4.10 Future directions

To address this study’s limitations and mitigate the growing burden of NTM infections in China, future research should prioritize coordinated efforts across key domains.

Standardizing and advancing diagnostics through multi-center studies employing uniform molecular methods (such as tNGS and WGS) is critical. Integrating molecular, proteomic, and sequencing technologies will reduce methodological bias, enhance diagnostic accuracy, expand NTM species coverage, and develop accessible, cost-effective tools—resolving current research inconsistencies and improving epidemiological result reliability.

Establishing a graded monitoring network that integrates clinical, environmental, and microbiome data will strengthen surveillance capacity. This network should extend to underrepresented rural/remote regions, clarify drivers of NTM prevalence and distribution via cross-domain data integration, and enhance cross-border surveillance for precise pathogen tracing—aligning with tuberculosis elimination goals and supporting targeted control measures. Advancing clinical management entails evaluating novel antibacterial agents (e.g., amikacin liposome, omadacycline), designing individualized regimens via molecular DST—particularly for resistant strains like the MABC—and investigating host-immune-mycobacterial virulence interactions to inform targeted interventions. We recommend that future studies adopt more flexible yet rigorous criteria for including Taiwanese literature to enhance the representativeness of data from this region, complementing broader efforts to strengthen regional coverage. Three aspects—climatic factor validation, diagnostic framework adaptation, and socioeconomic gradient analysis—will be critical components for in-depth discussion in future research focusing on comparing the epidemiological trends of NTM in China with global epidemiological patterns. Collectively, these efforts will establish a unified, standardized NTM surveillance and control system in China, supporting long-term mitigation of NTM-related public health impact.

## Data Availability

The original contributions presented in the study are included in the article/Supplementary material, further inquiries can be directed to the corresponding author.
